# Usefulness of assessment of the Clinical Frailty Scale and the Dementia Assessment Sheet for Community-based Integrated Care System 21-items at the time of initiation of maintenance hemodialysis in older patients with chronic kidney disease

**DOI:** 10.1371/journal.pone.0301715

**Published:** 2024-05-23

**Authors:** Seiji Hashimoto, Mitsuyo Itabashi, Kenta Taito, Ayano Izawa, Yui Ota, Takaaki Tsuchiya, Shiho Matsuno, Masahiro Arai, Noriko Yamanaka, Takako Saito, Masatoshi Oka, Noriyuki Suzuki, Yuki Tsuruta, Takashi Takei

**Affiliations:** 1 Department of Nephrology and Dialysis, Tokyo Metropolitan Institute for Geriatrics and Gerontology, Itabashi, Tokyo, Japan; 2 Department of Geriatric Medicine, The University of Tokyo, Bunkyo, Tokyo, Japan; 3 Department of Nephrology, Tokyo Medical University, Shinjuku, Tokyo, Japan; 4 Keitenkai, Tsuruta Itabashi Clinic, Kita, Tokyo, Japan; Instituto Nacional de Geriatria, MEXICO

## Abstract

**Introduction:**

We examined whether the Clinical Frailty Scale (CFS), a widely adopted tool for stratifying the degree of frailty, and the Dementia Assessment Sheet for Community-based Integrated Care System 21-items (DASC-21), a simple tool for simultaneous assessment of impaired cognition and impaired ADL, at the time of initiation of hemodialysis is useful tool of older patients for the outcome and prognosis.

**Methods:**

Data for 101 patients aged 75 years or older (mean age, 84.3 years) with ESRD who were initiated on hemodialysis and could be followed up for a period of 6 months were reviewed.

**Results:**

The 6-month survival curves showed a significantly higher number of deaths in the frailty (CFS≥5) group than in the normal to vulnerable (CFS<5) group (p<0.01). The CFS level was also significantly higher (6.5±1.5) in patients who died within 6 months of dialysis initiation as compared with that (4.6±1.7) in patients who survived (p<0.01). On the other hand, the total score of DASC-21 was related to need for inpatient maintenance dialysis (p<0.01). The total score on the DASC-21 were found as showing significant correlations with the CFS level. The IADL outside the home was identified in the DASC-21 sub-analyses as being correlated with CFS.

**Conclusions:**

The CFS and the DASC-21 appeared to be a useful predictive tool of outcome and prognosis for older patients being initiated on hemodialysis. Assessment by the CFS or the DASC-21 might be useful for selecting the renal replacement therapy by shared decision-making and for advance care planning.

## Introduction

The rates of initiation of maintenance hemodialysis are increasing at a faster rate in older adults aged 75 years or over than in younger age groups. According to a nationwide survey conducted by the Japan Society for Dialysis Therapy, the mean age at initiation of maintenance dialysis was 68.7 years in 2013 [1], but increased steadily thereafter to reach 70.8 years in 2020 [2]. Dialysis patients were prone to frailty, which accounts for 67.7% of all dialysis patients, and especially in the older over 75 years of age, it was reported to be about 80% [3]. It has also been shown that there is a progressive decline in physical function after the introduction of dialysis [4]. Dialysis patients are also known to have 2.57 times the risk of dementia as non-dialysis patients [5]. Some reports suggest that there is no clear difference in survival between induction and non-induction of dialysis in patients over 75 years of age who need care [6–8]. Therefore, some reports suggest that conservative renal management (CKM) without dialysis may be preferable in older CKD patients with frailty, impaired daily functioning, and cognitive decline [9, 10]. This means that the assessment of frailty and daily functioning as well as cognitive function before deciding to start dialysis is of critical importance. The Clinical Frailty Scale (CFS) was reported as frailty scale as it has been validated for hospitalized older adults, is easy to use and has good inter-rater reliability, and correlates well with the Cardiovascular Health Study (CHS) Index and the Frailty Index [11, 12]. It has also been shown to retain validity when retrospectively applied [13, 14]. The Dementia Assessment Sheet for Community-based Integrated Care System 21-items; DASC-21 [15] was developed as a simple tool to simultaneously assess impaired cognitive functions and impaired ADL and has been shown to be useful in a comprehensive geriatric assessment (CGA) assessment. The DASC-21 correlates significantly with the Mini-Mental State Examination (MMSE) and Frontal Assessment Battery (FAB) and has been validated as a reliable tool for the assessment of cognitive impairment and impairment of ADL [16, 17]. Because the DASC-21 includes questions on IADL, basic ADL (BADL), and cognition, it may be useful for classifying older CKD patients with cognitive impairment and frailty into appropriate categories. Therefore, the purpose of this study was to evaluate whether the assessment of cognitive functions and frailty at the time of initiation of maintenance dialysis might be useful for predicting the outcome and prognosis in older patients and contribute to decision-making on initiation of renal replacement therapy.

## Methods

### Study design and patients

Among the patients admitted to the Department of Nephrology and Dialysis in the hospital of the Tokyo Metropolitan Institute for Geriatrics and Gerontology between 2019 and 2021, and introduced to hemodialysis, the 101 patients aged 75 years or older (mean age, 84.3 years) with end-stage renal disease (ESRD) who could be followed up for a period of 6 months and over were enrolled. The prognostic impact of frailty, daily functioning, and cognitive function at the time of hemodialysis initiation in the subjects was investigated retrospectively through chart review. The subjects were assessed by CFS, a widely adopted tool for stratifying the degree of frailty, and the DASC-21, an assessment tool for cognitive and impaired ADL, at the time of their admission for initiation of hemodialysis. The patients’ data were used anonymously, in compliance with the latest version of the Declaration of Helsinki. This study was conducted with the approval of the Institutional Review Board of the relevant medical institution (approval number: R22-017) and with the patient’s informed consent and written consent with data accessed after June 28, 2022.

### CFS and DASC21

CFS is not a questionnaire, but a way to summarize information from a clinical encounter and roughly quantify an individual’s overall health status. The patients were divided 9 groups according to a visual and descriptive scale [11]. Classification was considered normal to vulnerable, “non-frail group” for a CFS score of <5, and “frail group” for a CFS score of ≥5 [11]. The DASC-21 consists of 21 items categorized into three domains: cognitive functions (9 items), IADL (6 items), and BADL (6 items) [15] (Table 1). The items are rated on a 4-point Likert scale from 1 (“intact”) to 4 (“severe impairment”), with the total score ranging from 21 to 84. High scores indicate poor functions. A total score of ≥31 on the DASC-21 indicates a “risk of dementia”. According to the score on DASC-21, the patients were divided into two groups: the group with a total score of <31 was categorized into the “normal group,” and the group with a score of >31 was categorized into the “dementia-risk group”. In addition, the dementia-risk group was further subdivided into three groups (groups with a risk of low-level dementia, medium level dementia, and serious level dementia) according to a classification reported in a previous study [15, 16].

**Table 1:** The Dementia Assessment Sheet for Community-based Integrated Care System—21 items (DASC-21).

		1 point	2 points	3 points	4 points	Topic		Remarks
A	Do you have the impression that he/she forgets a lot of things?	a. No	b. Yes, a little	c. Yes	d. Yes, a lot	Introductory questions (no points)		
B	Compared to last year, do you have the impression that he/she forgets more things?	a. No	b. Yes, a little	c. Yes	d. All the time			
1	Does he/she forgets where he/she puts things such as his/her wallet or keys?	a. Never	b. Sometimes	c. Frequently	d. Always	Memory	Recent memory	
2	Does he/she forgets a conversation that happened 5 minutes ago?	a. Never	b. Sometimes	c. Frequently	d. Always			
3	Does he/she forgets his/her own birth date?	a. Never	b. Sometimes	c. Frequently	d. Always		Remote memory	
4	Does he/she forgets what day and month it is?	a. Never	b. Sometimes	c. Frequently	d. Always	Orientation	Time	
5	Does he/she forgets where he/she is?	a. Never	b. Sometimes	c. Frequently	d. Always		Space	
6	Does he/she forgets how to get back home?	a. Never	b. Sometimes	c. Frequently	d. Always		Route finding	
7	When the supply of electricity, gas or water ceases, can he/she deals appropriately with the issue?	a. Yes, without difficulty	b. Can most of the time	c. Can’t most of the time	d. Not at all	Solving issues/ Common sense	Solving issues	
8	Can he/she makes plans for the day?	a. Yes, without difficulty	b. Can most of the time	c. Can’t most of the time	d. Not at all			
9	Can he/she selects his/her own clothes appropriately according to the season or the situation?	a. Yes, without difficulty	b. Can most of the time	c. Can’t most of the time	d. Not at all		Social common sense	
10	Can he/she buys things by himself/herself?	a. Yes, without difficulty	b. Can most of the time	c. Can’t most of the time	d. Not at all	IADL outside the home	Shopping	
11	Can he/she uses the bus, the train or a car by himself/herself?	a. Yes, without difficulty	b. Can most of the time	c. Can’t most of the time	d. Not at all		Transportation	
12	Can he/she pays the rent and bills, withdraw money or make a deposit by himself/herself?	a. Yes, without difficulty	b. Can most of the time	c. Can’t most of the time	d. Not at all		Money management	
13	Can he/she makes phone calls?	a. Yes, without difficulty	b. Can most of the time	c. Can’t most of the time	d. Not at all	IADL inside the home	Phone calls	
14	Can he/she prepares food by himself/herself?	a. Yes, without difficulty	b. Can most of the time	c. Can’t most of the time	d. Not at all		Preparing food	
15	Can he/she takes the correct quantity of medication at the right time of the day?	a. Yes, without difficulty	b. Can most of the time	c. Can’t most of the time	d. Not at all		Medication	
16	Can he/she takes a bath by himself/herself?	a. Yes, without difficulty	b. Needs supervision or instructions	c. Needs partial assistance	d. Needs full assistance	Physical ADL (1)	Bathing	
17	Can he/she changes clothes by himself/herself?	a. Yes, without difficulty	b. Needs supervision or instructions	c. Needs partial assistance	d. Needs full assistance		Dressing	
18	Can he/she uses the toilet by himself/herself?	a. Yes, without difficulty	b. Needs supervision or instructions	c. Needs partial assistance	d. Needs full assistance		Using the toilet	
19	Can he/she takes care of his/her own appearance?	a. Yes, without difficulty	b. Needs supervision or instructions	c. Needs partial assistance	d. Needs full assistance	Physical ADL (2)	Grooming	
20	Can he/she eats on his/her own?	a. Yes, without difficulty	b. Needs supervision or instructions	c. Needs partial assistance	d. Needs full assistance		Eating	
21	Can he/she moves around the house by himself/herself?	a. Yes, without difficulty	b. Needs supervision or instructions	c. Needs partial assistance	d. Needs full assistance		Mobility	
								Total score: / 84 points

1)A total score of 31 or above on the DASC-21 indicates a “risk of dementia”.

2)A total score of 31 above and scores of 2 or below on all the items on remote memory, space orientation, social common sense and physical ADL indicate a “risk of low-level dementia”.

3)A total score of 31 above and a score of 3 or above on at least one (but not all) of the items on remote memory, space orientation, social common sense and physical ADL indicate a “risk of medium level dementia”.

4)A total score of 31 above and scores of 3 or above on all the items on remote memory, space orientation, social common sense and physical ADL indicate a “risk of serious level dementia”.

### Data collection

The baseline demographic data included the age, sex, smoking status, and living situation. The following clinical information about the subjects was collected from their medical charts: medical history (presence/absence of hypertension, diabetes mellitus, heart disease, cerebral vascular disease, cancer, and/or peripheral vascular disease); data on the systolic blood pressure (SBP), diastolic blood pressure (DBP), body mass index (BMI), serum hemoglobin (Hb), serum albumin (Alb), serum C-reactive protein (CRP), serum total cholesterol (TC), serum triglycerides (TG), hemoglobin A1c (HbA1c), blood urea nitrogen (BUN), serum creatinine (Cr), serum uric acid (UA), serum sodium (Na), serum potassium (K), serum calcium (Ca), serum phosphorus (P), serum intact-parathyroid hormone (i-PTH), serum brain natriuretic peptide (BNP), and the cardiothoracic ratio (CTR) on the x-ray. Furthermore, we examined the duration of hospitalization, death during hospitalization, and death within 6 months, and whether the patients were discharged home and started on outpatient maintenance dialysis, started on outpatient maintenance dialysis while residing in a nursing home, or transferred to the hospital for inpatient maintenance dialysis.

### Outcomes and prognosis

Outcome was defined as discharge home to outpatient maintenance dialysis, transfer to inpatient maintenance dialysis, nursing home admission to outpatient maintenance dialysis, or death during hospitalization, and prognosis was defined as survival and death within 6 months of induction of dialysis. Duration of hospitalization was also noted. We examined the outcome and prognosis for differences with and without frailty (CFS: ≥5) and dementia-risk (DASC-21: ≥31). Sub-analysis was conducted to determine which DASC-21 items (memory, orientation, solving issue/common sense, IADL outside the home, IADL inside the home, physical ADL1, 2) were relevant. We investigated the discharge outcomes and prognosis of older CKD patients after induction of hemodialysis.

### Statistical analysis

Data are expressed as the means ± standard deviation (SD). All the analyzed variables were tested for distribution. To analyze the statistical significance of differences in the data, a *t*-test and ANOVA were used for data showing normal distribution, while the Mann–Whitney U-test was used for data showing a skewed distribution. The distribution of each indicator was examined before the statistical tests. Categorical data were analyzed using the χ^2^ test. The statistical significances of the correlations among the groups were analyzed by calculating the Pearson correlation coefficients or the point bi-serial correlation coefficients. The analysis was performed for CFS and DASC-21 as separate models. Multivariate analysis was performed with dependent variables as factors related to outcome and prognosis that were significantly different in the univariate analysis and CFS as the independent variable. The same analysis was performed with DASC-21 as the independent variable. If a correlation was found between CFS and DASC-21 scores, the relationship between CFS and each item on the DASC-21 was also examined. Receiver operating characteristic (ROC) curve analysis was carried out to examine death within 6 months based on CFS or DASC-21. *P* <0.05 was considered as denoting statistical significance. Survival was assessed by the Kaplan-Meier method. The analyses were performed using the JMP 15 software (SAS Institute, Cary, NC).

## Results

### Outcome after the introduction of hemodialysis

There were 24 deaths (23.8%) within 6 months in this study. The average length of hospitalization was 56±32 days, and discharge outcomes were death during hospitalization in 14 patients (13.9%), inpatient maintenance dialysis after transferrin 19 patients (18.8%), outpatient maintenance dialysis while residing in a nursing home in 4 patients (4%), and discharged home and outpatient maintenance dialysis in 63 patients (62.4%). Deaths during hospitalization were due to infection in 7 patients (50.0%), cardiovascular disease in 4 patients (28.6%), cancer 2 patient (14.3%), and cerebrovascular disease in 1 patient (7.1%).

### Efficacy of the CFS

**Fig 1:**
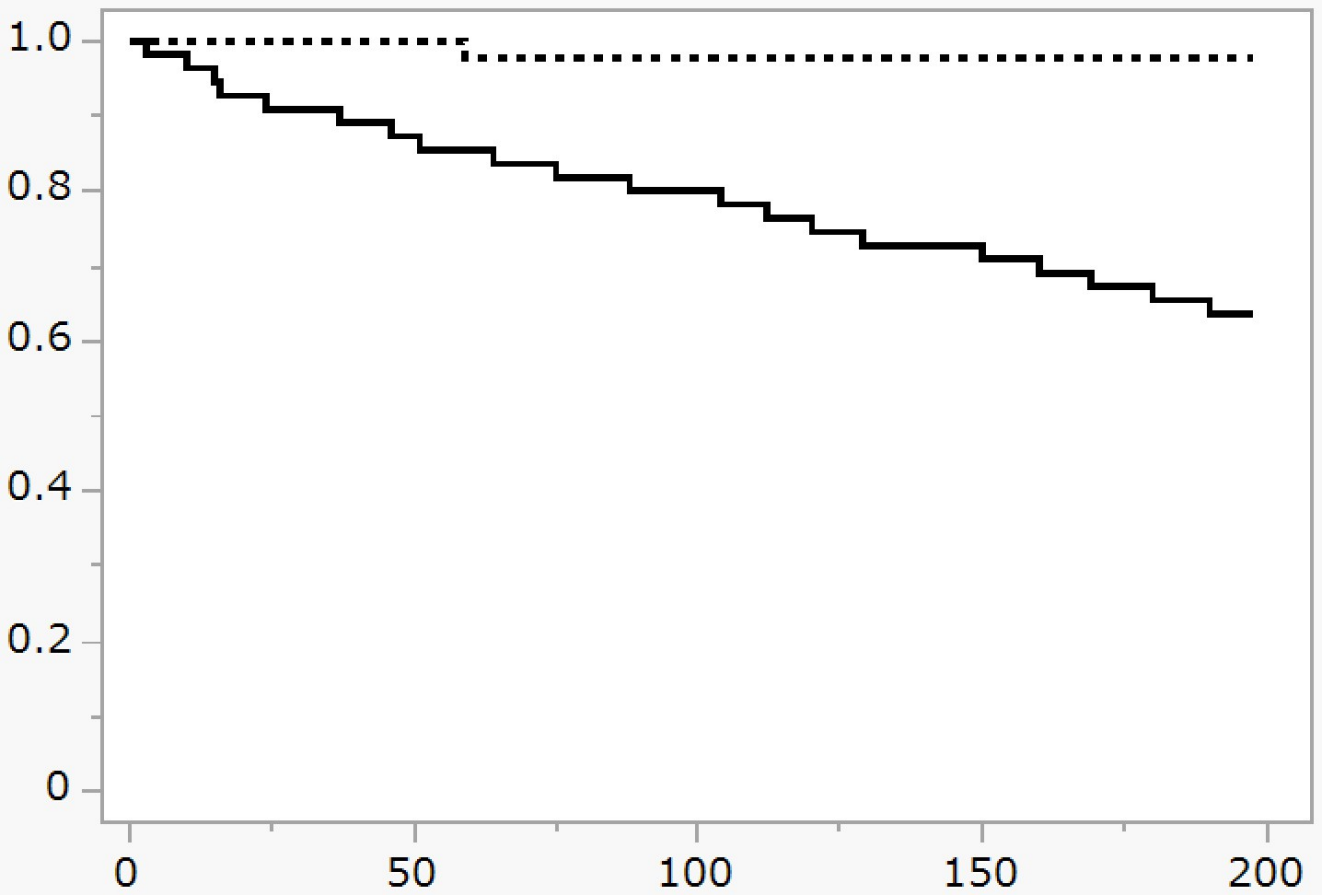
Kaplan–Meier analysis of survival in the group with a total score of ≥5 on the CFS, indicative of “frailty”. Comparison of the survival rate between the frail group (dotted line) and the non-frail group (solid line). The X-axis indicates the follow-up period (days), and the Y-axis indicates the survival rate (%). p <0.001 between the frailty and normal groups as assessed by the log-rank test.

The mean score on the CFS was 5.0±1.8 (CFS: n = 0, CFS2: n = 1, CFS3: n = 33, CFS4: n = 11, CFS5: n = 10, CFS6: n = 15, CFS7: n = 22, CFS8: n = 9, CFS9: n = 0). The 9-points scale was divided into a non-frail group (CSF<5: n = 45 (44.6%)) and a frail group (CFS≥5: n = 56 (55.4%)). Comparison of the two groups revealed a significantly higher mean age (p<0.01), CRP (p<0.05), BNP (p<0.05), CTR (p<0.01) and duration in hospitalization (p<0.001), and a significantly higher number of death within 6 months after dialysis induction (p<0.001), death during hospitalization (p<0.001), patients transferred to the hospital for inpatient dialysis (p<0.001), emergency dialysis induction using dialysis catheter (p<0.05), cardiovascular disease(p<0.005), cerebral vascular disease (p<0.05) in the frail group. In contrast, there were significantly lower mean SBP (p<0.01), DBP (p<0.05), Hb (p<0.05) and Alb (p<0.01), and significantly fewer patients who were discharged home and became outpatients (p<0.001) and planned dialysis induction using arteriovenous fistula (p<0.001) in the frail group (Table 2). The following factors in prognosis and outcome were found as showing significant correlations with the score of CFS: inpatient maintenance dialysis after transfer, discharged home and outpatient maintenance dialysis, duration of hospitalization, and death within 6 months (p<0.001, p<0.001, p<0.001, and p<0.001, respectively). (Table 3). In multivariate analysis, death within 6 months (p<0.005) was significantly associated with CFS (Table 4). The 6-month survival curves showed a significantly higher number of deaths in the frail group than in the non-frail group (p<0.001). (Fig 1). The CFS level was significantly higher in patients who died within 6 months of dialysis initiation (6.5±1.5) as compared with that in patients who survived (4.6±1.7)(p<0.001). The cut-off of score on the CFS for death within 6 months was 6.0, and the areas under the curve (AUC) was 0.78 (S1 Fig). The sensitivity and specificity of the CFS for death within 6 months were 79% and 56%, respectively.

**Table 2:** Clinical data at the baseline of the overall subject population and comparison of the clinical features in the CFS and DASC-21.

	Overall	Clinical Frailty Scale			DASC-21					
		Non-frail	frail	p value	Normal*^+^	Dementia-risk			p value*	p value^+^
						Overall*	Mild dementia^+^	Medium and serious dementia^+^		
Number(N)	101	45	56		55	46	18	28		
Age(Y)	83.4±5.0	81.7±4.1	84.8±5.2	<0.01	81.5±4.1	85.7±5.1	85.7±5.0	85.7±5.3	<0.001	<0.001
Female(N)	52	19	33	n.s.	27	25	8	17	n.s.	n.s.
Death within 6 months after dialysis induction(N)	24	3	21	<0.001	10	14	3	11	n.s.	n.s.
Duration in hospitalization (Day)	35.5±35.5	17.4±13.0	51.0±41.2	<0.001	30.2±32.3	41.8±39.0	32.1±24.9	48.6±45.7	n.s.	n.s.
Death during hospitalization(N)	14	1	13	<0.001	6	8	1	7	n.s.	n.s.
Inpatient maintenance dialysis after transfer(N)	18	1	17	<0.001	5	13	5	8	<0.01	<0.05
Outpatient maintenance dialysis while residing in a nursing home(N)	4	0	4	n.s.	1	3	1	2	n.s.	n.s.
Discharged home and outpatient maintenance dialysis(N)	63	43	20	<0.001	43	20	11	9	<0.001	<0.001
Planned dialysis induction using arteriovenous fistula(N)	59	36	23	<0.001	33	26	13	13	n.s.	n.s.
Emergency Dialysis induction using dialysis catheter(N)	42	12	30	<0.05	22	20	6	14	n.s.	n.s.
Body mass index(kg/m2)	22.7±4.1	23.1±3.8	22.4±4.3	n.s.	22.4±3.6	23.1±4.6	23.9±1.0	22.5±4.4	n.s.	n.s.
Systolic blood pressure(mmHg)	148.8±23.7	155.7±25.4	143.2±20.8	<0.01	151.1±24.9	146.0±22.2	141.3±23.4	149.8±21.0	n.s.	n.s.
Diastolic blood pressure(mmHg)	72.6±13.1	75.6±14.1	70.1±11.8	<0.05	72.6±13.7	72.6±12.6	71.2±11.3	73.7±13.7	n.s.	n.s.
Hemoglobin(g/dL)	9.7±1.4	10.0±1.5	9.4±1.3	<0.05	9.7±1.5	9.6±1.2	9.7±1.0	9.6±1.4	n.s.	n.s.
Albumin(g/dL)	2.9±0.6	3.0±0.5	2.7±0.5	<0.01	2.9±0.6	2.8±0.5	2.8±0.5	2.7±0.5	n.s.	n.s.
C-reactive protein(mg/dL)	2.4±3.8	1.4±2.9	3.2±4.3	<0.05	2.7±4.3	2.1±3.2	1.9±3.4	2.2±3.1	n.s.	n.s.
Blood urea nitrogen(mg/dL)	81.0±24.3	78.2±21.9	83.3±26.0	n.s.	77.5±21.9	85.4±26.2	88.7±26.7	84.7±26.1	n.s.	n.s.
Creatinine (mg/dL)	6.4±2.0	6.7±2.0	6.3±2.0	n.s.	6.6±2.1	6.0±1.7	6.4±1.4	5.8±1.9	n.s.	n.s.
Uric acid(mg/dL)	7.2±2.2	7.3±1.9	7.1±2.5	n.s.	7.0±2.0	7.5±2.5	7.6±2.6	7.4±2.5	n.s.	n.s.
Sodium(mEq/L)	138.2±4.7	138.1±3.3	138.2±5.6	n.s.	137.7±4.2	138.7±5.3	137.9±4.8	139.3±5.6	n.s.	n.s.
Potassium(mEq/L)	4.4±0.8	4.4±0.7	4.5±0.9	n.s.	4.5±0.8	4.4±0.9	4.3±0.9	4.4±0.9	n.s.	n.s.
Calcium(mg/dL)	7.9±0.9	8.1±0.8	7.9±0.9	n.s.	7.9±0.8	7.8±0.9	7.9±0.6	7.8±1.1	n.s.	n.s.
Phosphorus (mg/dL)	5.5±1.6	5.5±1.7	5.5±1.6	n.s.	5.4±1.7	5.6±1.6	5.8±1.7	5.5±1.5	n.s.	n.s.
Intact-parathyroid hormone (pg/mL)	258.8±400.1	276.0±120.8	250.0±100.6	n.s.	198.1±117.1	335.8±583.0	262.7±173.9	387.6±750.5	n.s.	n.s.
Total cholesterol(mg/dL)	164.4±39.5	164.4±39.6	164.4±39.7	n.s.	167.0±44.8	161.5±32.7	168.9±30.9	156.4±33.5	n.s.	n.s.
Triglyceride (mg/dL)	114.1±47.8	120.9±53.5	108.8±42.5	n.s.	121.3±50.4	105.8±43.8	104.9±41.4	106.3±46.1	n.s.	n.s.
Hemoglobin A1c (NGSP) (%)	6.0±1.8	5.8±0.6	6.0±1.10	n.s.	6.2±2.4	5.7±0.8	5.8±0.9	5.7±0.7	n.s.	n.s.
Brain natriuretic peptide	488.1±657.1	313.5±365.5	624.8±793.6	<0.05	522.6±779.0	448.3±486.9	388.1±329.1	502.4±598.5	n.s.	n.s.
Cardio-thoracic ratio (%)	56.6±7.9	54.1±6.6	58.6±8.3	<0.01	54.1±6.4	59.5±8.6	59.8±9.6	59.3±8.0	<0.001	<0.005
Cardiovascular disease(N)	44	13	31	<0.005	23	21	7	14	n.s.	n.s.
Cerebral vascular disease(N)	20	5	15	<0.05	7	13	3	10	n.s.	n.s.
Hypertension(N)	99	43	56	n.s.	54	45	19	26	n.s.	n.s.
Diabetes mellitus(N)	54	22	32	n.s.	34	20	5	15	n.s.	n.s.
Peripheral vascular disease(N)	16	6	10	n.s.	9	7	1	6	n.s.	n.s.
Cancer(N)	21	8	13	n.s.	8	13	5	8	n.s.	n.s.
Smoker (Brinkman index)	397.2±534.0	410.0±533.7	384.6±544.9	n.s.	488.9±607.9	281.5±400.5	179.4±296.2	358.1±454.7	n.s.	n.s.
Number of cohabitants(N)	2.1±1.1	2.1±1.3	2.0±0.8	n.s.	1.9±1.1	2.2±1.1	2.5±1.5	2.1±0.7	n.s.	n.s.
CFS	5.0±1.9	3.2±0.5	6.5±1.0	p<0.001	4.2±1.7	6.0±0.5	5.2±1.5	6.6±1.3	<0.001	<0.005
DASC score	34.7±15.0	26.7±6.9	41.1±16.7	p<0.001	24.4±3.2	47.0±14.1	37.9±7.2	53.4±14.4	<0.001	<0.001

Values are expressed as mean±SD,

* p<0.05,

n.s.; no significance

**Table 3:** Correlation on outcomes between the total score on the CFS and various items determined by univariate analysis.

item	R^2^	p value
Death within 6 months after dialysis induction(N)	0.18	<0.001*
Duration in hospitalization (Day)	0.23	<0.001*
Death during hospitalization(N)	0.29	<0.001*
Inpatient maintenance dialysis after transfer(N)	0.15	<0.001*
Outpatient maintenance dialysis while residing in a nursing home(N)	0.08	n.s.
Discharged home and outpatient maintenance dialysis(N)	0.4	<0.001*

* p<0.05,

n.s.; no significance

**Table 4:** Multivariate analysis was performed to evaluate the contributors to the total score on the CFS.

item	β	F value	t value	CI lower95%	CI upper95%	p value
Death within 6 months after dialysis induction(N)	-0.58	8.13	-2.85	-1.00	-0.17	<0.005*
Discharged home and outpatient maintenance dialysis(N)	0.35	4.38	1.86	0.25	0.86	n.s.
Duration in hospitalization (Day)	0.01	2.36	1.54	0.00	0.02	n.s.
Inpatient maintenance dialysis after transfer(N)	-0.12	0.15	-0.38	-0.76	-0.51	n.s.
Death during hospitalization(N)	0.10	0.06	0.25	-0.65	0.84	n.s.

* p<0.05,

n.s.; no significance

### Efficacy of the DASC-21

The mean score on the DASC-21 was 34.7±15.0. The results of the sub-analysis were as follows: memory 4.3±1.7 score, orientation 4.0±1.9 score, solving issue/common sense 5.4±2.7 score, IADL outside the home 6.6±3.5 score, IADL inside the home 5.4±2.8 score, physical ADL1 4.7±2.9 score, and ADL2 4.1±2.1 score. There were 46 patients (45.5%) in the dementia-risk group, with a mean DASC-21 score of 47.0±14.1. The dementia-risk group was further subdivided into a group with a risk of low level dementia (n = 19 (41.3%)), a group with a risk of medium level dementia (n = 26 (56.5%)), and a group with a risk of serious level dementia (n = 1 (0.2%)). Comparison of the normal and dementia-risk groups revealed a significantly higher mean age (p<0.001), a significantly higher number of patients transferred to the hospital for inpatient dialysis (p<0.01), significantly fewer patients who were discharged home and became outpatients (p<0.001), and a significantly higher number of patients with an elevated CTR (p<0.001) in the dementia-risk group (Table 2). The analyses were conducted to compare between three groups: the normal group, the group with a risk of low level dementia and the group with a risk of medium/serious level dementia. There were a significantly differences in age (p<0.001), discharged home and became outpatients (p<0.001), number of patients transferred to the hospital for inpatient dialysis (p<0.05), and CTR (p<0.005) (Table 2).

In addition, factors according to prognosis and outcome correlated with the total scores on DASC-21 were also examined. The following factors were found as showing significant correlations with the total score on the DASC-21: people needing inpatient maintenance dialysis after transfer, duration of hospitalization, outpatient maintenance dialysis while residing in a nursing home and discharged home and outpatient maintenance dialysis (p<0.001, p<0.05, p<0.05, and p<0.001, respectively) (Table 5). In multivariate analysis, the need for inpatient maintenance dialysis after transfer (p<0.05) was significantly associated with DASC-21 (Table 6). The DASC-21 sub-analysis identified memory (p<0.05), solving issues/common sense (p<0.05), IADL outside the home (p<0.005), IADL inside the home (p<0.005), physical ADL1 (p<0.001) and physical ADL2 (p<0.001) as being correlated with the need for inpatient maintenance dialysis after transfer (S1 Table). Multivariate analysis identified physical ADL2 (p<0.05) as independent contributing factor for the need for inpatient maintenance dialysis after transfer (S2 Table).

**Table 5:** Correlation between the total score on the DASC-21 and outcomes determined by univariate analysis.

item	R^2^	p value
Death within 6 months after dialysis induction(N)	0.02	n.s.
Duration in hospitalization (Day)	0.07	<0.05*
Death during hospitalization(N)	0.01	n.s.
Inpatient maintenance dialysis after transfer(N)	0.11	<0.001*
Outpatient maintenance dialysis while residing in a nursing home(N)	0.11	<0.05*
Discharged home and outpatient maintenance dialysis(N)	0.12	<0.001*

* p<0.05,

n.s.; no significance

**Table 6:** Multivariate analysis was performed to evaluate the contributors to the total score on the DASC-21.

items	β	F value	t value	CI lower95%	CI upper95%	p value
Inpatient maintenance dialysis after transfer(N)	-4.99	4.49	-2.12	-9.65	-0.32	<0.05*
Outpatient maintenance dialysis while residing in a nursing home(N)	-8.11	3.39	-1.82	-15.79	-0.43	n.s.
Duration in hospitalization (Day)	0.05	1.50	1.22	-0.03	0.14	n.s.
Discharged home and outpatient maintenance dialysis(N)	1.95	0.80	0.89	-2.37	6.26	n.s.

* p<0.05,

n.s.; no significance

### Relation between the CFS and the DASC-21

**Fig 2:**
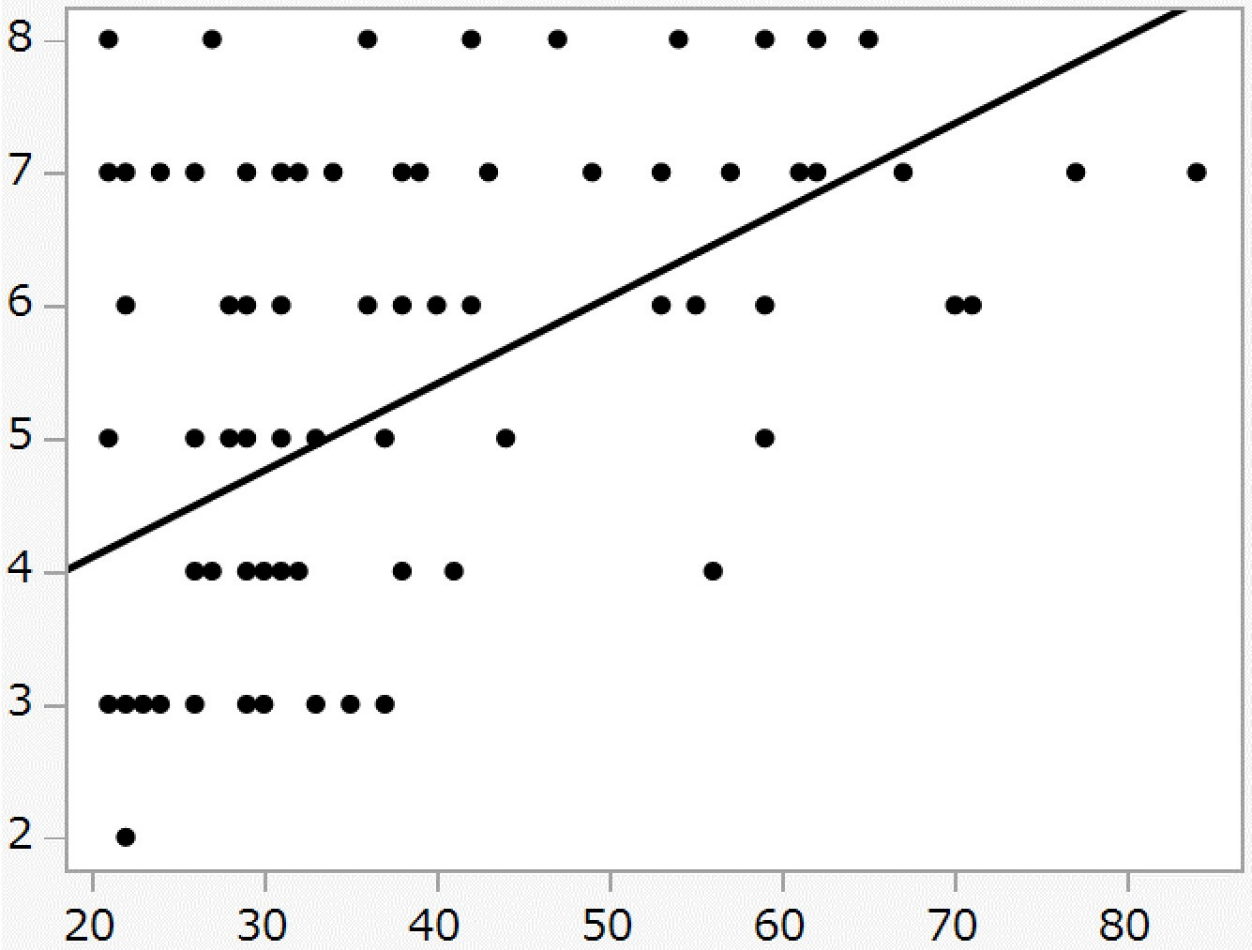
Relation between the score of CSF and the score of DASC-21. The score of DASC-21(the X-axis) was significantly correlated with the score of CFS (the Y-axis) (r = -0.45 (p < 0.01).

The total score on the DASC-21 were found as showing significant correlations with the CFS level (p<0.001) (Fig 2). The following topics in the DASC-21 sub-analyses were found as showing significant correlations with the CFS: Solving issues/Common sense (p<0.005), IADL outside the home (p<0.001), IADL inside the home (p<0.001), physical ADL1 (p<0.001), and physical ADL2 (p<0.001) in (Table 7). In multivariate analysis, IADL outside the home (p<0.005) was significantly associated with CFS (Table 8).

**Table 7:** Correlations between the CFS and scores on various items of the DASC-21 determined by univariate analysis.

item	R^2^	p value
Memory	0.04	<0.05*
Orientation	0.09	<0.005*
Solving issues/ Common sense	0.12	<0.001*
IADL outside the home	0.32	<0.001*
IADL inside the home	0.26	<0.001*
Physical ADL1	0.30	<0.001*
Physical ADL2	0.27	<0.001*

* p<0.05

**Table 8:** Multivariate analysis was performed to evaluate the contributors to the CFS.

items	β	F value	t value	CI lower95%	CI upper95%	p value
IADL outside the home	0.21	7.50	2.74	0.06	0.36	<0.01*
Memory	-0.27	3.72	-1.93	-0.55	0.01	n.s.
Solving issues/ Common sense	-0.15	1.91	-1.38	-0.36	0.07	n.s.
IADL inside the home	0.17	1.63	1.28	-0.09	0.44	n.s.
Physical ADL1	0.19	1.6	1.26	-0.11	0.49	n.s.
Orientation	0.1	0.62	0.79	-0.15	0.36	n.s.
Physical ADL2	-0.01	0.01	-0.02	-0.40	0.39	n.s.

* p<0.05,

n.s.; no significance

Although we could not find a relationship between total scores on DASC-21 and death within 6 months after dialysis induction (Table 5), we found a relationship between IADL outside the home and death 6 months (R^2^ = 0.05, p<0.05). Between death and no-death within 6 months in the total score on DASC-21, there were also no significant difference (40.05±15.48 vs 30.25±14.41). However, mean the score of IADL outside the home was significantly higher in the group of death within 6 months in a two-group comparison of death and no-death within 6 months (8.38±3.67 vs 6.15±3.34, p<0.01). The ROC curve analysis showed a low AUC of 0.54 between the total scores on DASC 21 and the death within 6 months, while IADL outside the home had an AUC of 0.65, sensitivity of 58% and specificity of 70% at a cut-off value of 8.0 (S2 Fig).

## Discussion

Dialysis therapy is essential for life support in patients with ESRD. However, it is difficult to comprehensively predict the prognosis, including the ADL, quality of life, and duration of hospital stay in older patients with various comorbidities. The need for dialysis therapy thrice a week poses a heavy burden on older patients, and also their families and caregivers. Recently, there have been scattered reports on the possibility of forgoing dialysis, with conservative kidney management (CKM) [18] drawing attention as a new therapeutic option [19–24]. It has been suggested that CKM can be a useful alternative in patients over 80 years of age, with no difference in survival found in a previous study between a group that received CKM without dialysis and a group that received maintenance dialysis [25]. It has also been reported that while renal replacement therapy (RRT) is significantly better than CKM in terms of the survival [26], the quality of life tended to be similar or better in patients receiving CKM than in those receiving RRT [27, 28]. Currently, selection of RRT, that is, whether to initiate a patient on dialysis or not, is made by shared decision-making (SDM) between the patient, his/her family, and the medical team [29]. Although the role of SDM for older patients with advanced CKD is important, there is also a heavy psychological burden on the medical staff. The development of Advance Care Planning (ACP) [30], a process in which older renal failure patients discuss their future treatment and care with their families in advance to prepare for future decline in decision-making capacity, the decision-making process initiating versus not initiating dialysis, and the methodology for palliative care, are also urgent issues.

In this study, we investigated whether the CFS, a widely adopted tool for stratifying the degree of frailty [11–14], and the DASC-21, a simple scale to assess cognitive and functional decline [15, 16], could be used in older patients to predict the patient outcomes and prognosis at the time of initiation of dialysis.

Validation of CSF showed that frailty was significantly associated with life expectancy, with nearly half of the patients with frailty (CFS≥5) dying within 6 months. Previous reports have shown that frailty assessed in the Cardiovascular Health Study is associated with an increased risk of mortality in patients with advanced-stage CKD and those undergoing maintenance dialysis [31], and the present study also showed that patients with frailty were at a higher risk of death. These were useful information for SDM and renal replacement therapy selection. In the previous literature on CFS and dialysis, the mortality rate was 7.29% in patients with a mean age of 61 years and CFS ≥5 in 33% of patients [32], whereas in this study, the mean age was 84.3 years, 55.4% had CFS≥5, and the 6-month mortality rate was 23.8%. This is the first report showing a relationship between CFS and mortality in patients aged 75 years or older. There is also a report of progressive decline in physical function after the induction of dialysis in CKD patients in a nursing home with an average age of 73 years. According to this report, 58% of patients died at 12 months after induction, and only 13% of survivors maintained the same level of physical function as at induction [4]. In this study, 14 patients died during hospitalization after induction of dialysis, and 10 more died 6 months after discharge. According to a Norwegian report, the average length of hospital stay to start dialysis was 7 days (1–59 days) for CKD patients referred early to a nephrologist (mean age 56 years) and 31 days (7–73 days) for patients referred late (mean age 72 years) [33]. Although our study did not examine the timing of patient referral, the length of hospital stay was 22.6 days for discharge to home, while 54.7 days for transfer to a hospital or nursing home. The average length of stay for patients who died during hospitalization was 68.1 days. The main reason for the longer average length of hospital stay of 56 days might have been due to the patients’ old age and frailty. The results showed that higher DASC-21 scores were associated with an increased inpatient maintenance dialysis after transfer. The IADL outside the home on the DASC-21 showed strong correlations with the CFS. In DASC-21 sub-analysis, death within 6 months of initiation of dialysis was not associated with the scores on cognitive functions, such as disorientation, but was associated with the scores on the IADL outside the home. However, the IADL outside the home in the DASC-21 are simple assessments of "Can he/she buy things by him/herself?", "Can he/she use the bus, the train or a car by him/herself?", and "Can he/she pay the rent and bills, withdraw money or make a deposit by him/herself? The linkage between IADL outside the home and CFS in DASC 21 should be made with careful consideration.

The limitations of this study were that a CKM group was not included, and no long-term follow-up was performed. In addition, the study had a small sample size. This also limited the multiple comparisons.

Approximately 40% of patients with frailty died within 6 months, indicating that CKM may be a valid option for RRT in patients with high CFS scores.

In conclusion, the CFS and the DASC-21, a comprehensive geriatric assessment (CGA) scale, appears to be a useful prognostic and outcome tool for older patients being initiated on maintenance hemodialysis. Furthermore, assessment by these scales might also be useful for the selection of RRT by SDM and for ACP.

## Supporting information

S1 Table Correlations between the need of transfer for inpatient maintenance dialysis and scores on various items of the DASC-21 determined by univariate analysis. (DOCX)

S2 Table Multivariate analysis was performed to evaluate the contributors to the need of transfer for inpatient maintenance dialysis. (DOCX)

S1 Fig ROC curves of CFS scores for death within 6 months. (PDF)

S2 Fig ROC curves analysis of IADL outside of home scores on DASC-21 for death within 6 months. (PDF)
