# Supplementary material for: Usefulness of assessment of the Clinical Frailty Scale and the Dementia Assessment Sheet for Community-based Integrated Care System 21-items at the time of initiation of maintenance hemodialysis in older patients with chronic kidney disease
Source: PLoS One. 2024 May 23;19(5):e0301715. doi: 10.1371/journal.pone.0301715 (PMC11115207; doi:10.1371/journal.pone.0301715)
Supplement: S1 Table — (DOCX) [file pone.0301715.s001.docx]

**S1 Table:** Correlations between the need of transfer for inpatient maintenance dialysis and scores on various items of the DASC-21 determined by univariate analysis

|  |  |  |
| --- | --- | --- |
| item | R^2^ | p value |
| Memory | 0.06 | <0.05* |
| Orientation | 0.03 | n.s. |
| Solving issues/ Common sense | 0.06 | <0.05* |
| IADL outside the home | 0.10 | <0.005* |
| IADL inside the home | 0.10 | <0.005* |
| Physical ADL1 | 0.12 | <0.001* |
| Physical ADL2 | 0.17 | <0.001* |

*p<0.05, n.s.; no significance
