# Supplementary material for: Usefulness of assessment of the Clinical Frailty Scale and the Dementia Assessment Sheet for Community-based Integrated Care System 21-items at the time of initiation of maintenance hemodialysis in older patients with chronic kidney disease
Source: PLoS One. 2024 May 23;19(5):e0301715. doi: 10.1371/journal.pone.0301715 (PMC11115207; doi:10.1371/journal.pone.0301715)
Supplement: S2 Table — (DOCX) [file pone.0301715.s002.docx]

**S2 Table:** Multivariate analysis was performed to evaluate the contributors to the need of transfer for inpatient maintenance dialysis

|  | | |  | |  | |  |  | |  |
| --- | --- | --- | --- | --- | --- | --- | --- | --- | --- | --- |
| item | β | | | kai^2^ | | CI lower95% | | CI upper95% | p value | |
| Physical ADL2 | 0.77 | | | 3.65 | | 0.05 | | 1.65 | <0.05* | |
| Physical ADL1 | -0.37 | | | 1.54 | | -1.03 | | 0.16 | n.s. | |
| IADL outside the home | 0.14 | | | 0.88 | | -0.16 | | 0.43 | n.s. | |
| Solving issues/ Common sense | -0.06 | | | 0.08 | | -0.51 | | 0.32 | n.s. | |
| Memory | 0.04 | | | 0.02 | | -0.54 | | 0.56 | n.s. | |
| IADL inside the home | 0.02 | | | 0.01 | | -0.50 | | 0.52 | n.s. | |
| *p<0.05, n.s.; no significance | |  | | |  | |  |  | |  |
