# Supplementary figures and images for: Usefulness of assessment of the Clinical Frailty Scale and the Dementia Assessment Sheet for Community-based Integrated Care System 21-items at the time of initiation of maintenance hemodialysis in older patients with chronic kidney disease
Source: PLoS One. 2024 May 23;19(5):e0301715. doi: 10.1371/journal.pone.0301715 (PMC11115207; doi:10.1371/journal.pone.0301715)

## S1 Figure

ROC curves of CFS scores for death within 6 months

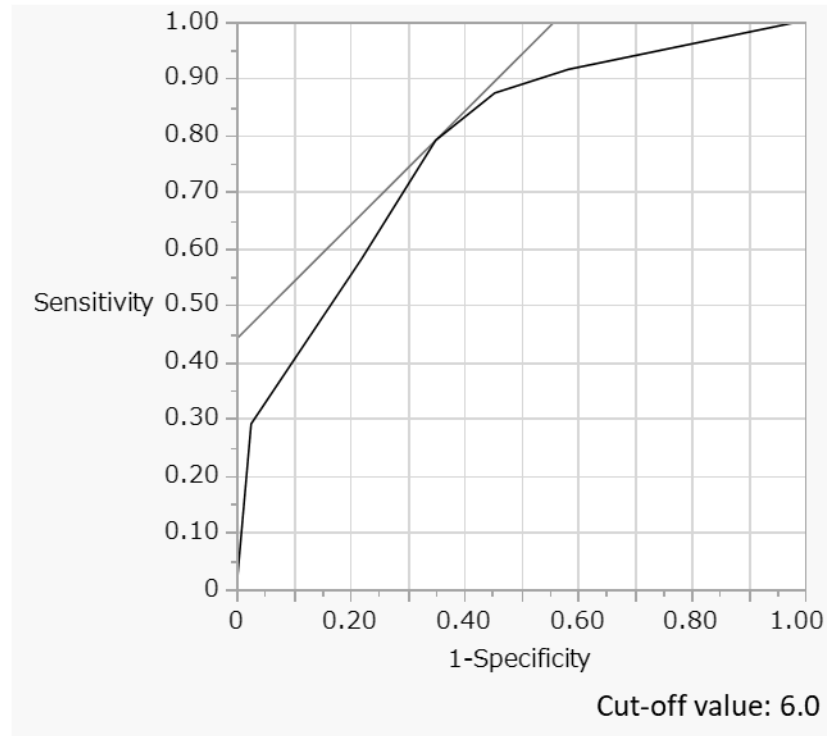

Supplement: S1 Fig — (PDF) [file pone.0301715.s003.pdf]

## S2 Figure

ROC curves analysis of IADL outside of home scores on DASC-21 for death within 6 months.

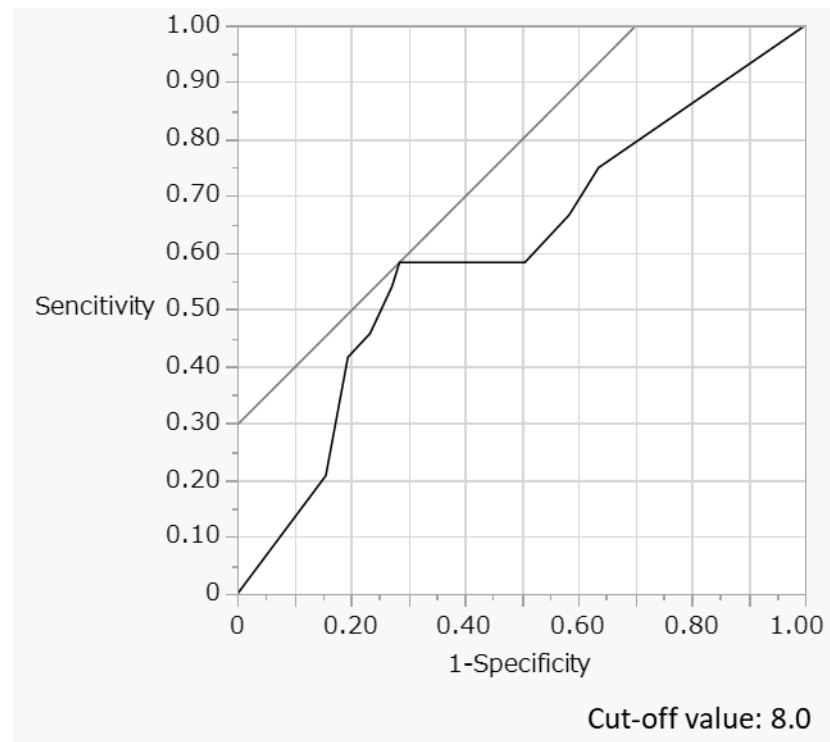

Supplement: S2 Fig — (PDF) [file pone.0301715.s004.pdf]
